# Outcomes based on plasma biomarkers in METEOR, a randomized phase 3 trial of cabozantinib vs everolimus in advanced renal cell carcinoma

**DOI:** 10.1186/s12885-021-08630-w

**Published:** 2021-08-07

**Authors:** Thomas Powles, Toni K. Choueiri, Robert J. Motzer, Eric Jonasch, Sumanta Pal, Nizar M. Tannir, Sabina Signoretti, Rajesh Kaldate, Christian Scheffold, Evelyn Wang, Dana T. Aftab, Bernard Escudier, Daniel J. George

**Affiliations:** 1 Barts Cancer Institute, Queen Mary University of London, London, UK; 2 Dana-Farber Cancer Center, Boston, MA USA; 3 Memorial Sloan Kettering Cancer Center, New York, NY USA; 4 University of Texas MD Anderson Cancer Center, Houston, TX USA; 5 City of Hope National Medical Center, Duarte, CA USA; 6 Brigham and Women’s Hospital, Boston, MA USA; 7 Exelixis, Inc, Alameda, CA USA; 8 Gustave-Roussy, Villejuif, France; 9 Duke Cancer Institute, Durham, NC USA

**Keywords:** Cabozantinib, Everolimus, Renal cell carcinoma, Biomarker, METEOR

## Abstract

**Background:**

In the phase 3 METEOR trial, cabozantinib improved progression-free survival (PFS) and overall survival (OS) versus everolimus in patients with advanced RCC after prior antiangiogenic therapy.

**Methods:**

In this exploratory analysis, plasma biomarkers from baseline and week 4 from 621 of 658 randomized patients were analyzed for CA9, HGF, MET, GAS6, AXL, VEGF, VEGFR2, and IL-8. PFS and OS were analyzed by baseline biomarker levels as both dichotomized and continuous variables using univariate and multivariable methods. For on-treatment changes, PFS and OS were analyzed using fold change in biomarker levels at week 4. Biomarkers were considered prognostic if *p* < 0.05 and predictive if p_interaction_ < 0.05 for the interaction between treatment and biomarker.

**Results:**

Hazard ratios for PFS and OS favored cabozantinib versus everolimus for both low and high baseline levels of all biomarkers (hazard ratios ≤0.78). In univariate analyses, low baseline HGF, AXL, and VEGF were prognostic for improvements in both PFS and OS with cabozantinib, and low HGF was prognostic for improvements in both PFS and OS with everolimus. Low AXL was predictive of relative improvement in PFS for cabozantinib versus everolimus. Results were generally consistent when baseline biomarkers were expressed as continuous variables, although none were predictive of benefit with treatment. In multivariable analysis, low baseline HGF was independently prognostic for improved PFS for both cabozantinib and everolimus; low HGF, GAS6, and VEGF were independently prognostic for improved OS with cabozantinib. No biomarkers were independently prognostic for OS with everolimus. On-treatment increases in some biomarkers appeared prognostic for PFS or OS with cabozantinib in univariate analyses; however, none were independently prognostic in multivariable analysis.

**Conclusions:**

PFS and OS were improved with cabozantinib versus everolimus at high and low baseline levels of all biomarkers. Low baseline HGF was consistently identified as a prognostic biomarker for improved PFS or OS with cabozantinib or everolimus, supporting further prospective evaluation of the prognostic significance of HGF in advanced RCC.

**Trial registration:**

ClinicalTrials.gov NCT01865747 (registered on 05/31/2013).

**Supplementary Information:**

The online version contains supplementary material available at 10.1186/s12885-021-08630-w.

## Background

The treatment landscape for advanced renal cell carcinoma (RCC) has vastly expanded in recent years [1, 2]. VEGF-targeted therapies, mTOR inhibitors, immune checkpoint inhibitors and combination therapies are all standard treatments that have shown improvements in outcome in phase 3 clinical trials. With the growing number of therapies, information on outcomes based on biomarkers may help with optimal therapy selection.

Cabozantinib is a standard of care for the treatment of advanced RCC that has shown efficacy in previously-treated patients and as a first-line therapy [3–5]. Cabozantinib inhibits multiple tyrosine kinases including MET, AXL, and VEGFR2 [6] that promote oncogenesis, angiogenesis, and resistance to antiangiogenic therapy in RCC. The VHL tumor suppressor gene is frequently inactivated in clear cell RCC, leading to hypoxia and upregulation of hypoxia-controlled genes including VEGF, MET, and AXL [7–10]. VEGF, MET, and AXL have also been associated with poor prognosis in RCC [9, 11, 12], and MET and AXL have been implicated in resistance to VEGFR-targeted therapy [13].

In the pivotal phase 3 METEOR trial, cabozantinib prolonged progression-free survival (PFS) and overall survival (OS) and increased the objective response rate (ORR) compared with the mTOR inhibitor everolimus in patients with advanced RCC after prior antiangiogenic therapy [3, 4]. Median PFS was 7.4 months with cabozantinib versus 3.9 months with everolimus (HR 0.51, 95% CI 0.41–0.61, *p* < 0.0001), and median OS was 21.4 months versus 16.5 months (HR 0.66, 95% CI 0.53–0.83, *p* = 0.0003) [3, 4]. Outcomes based on plasma biomarkers was an exploratory endpoint of the METEOR trial. Eight plasma proteins were evaluated for prognostic and predictive significance based on biological relevance and the target profile of cabozantinib: carbonic anhydrase 9 (CA9), hepatocyte growth factor (HGF), MET, GAS6, AXL, VEGF, VEGFR2, and IL-8. To evaluate these potential plasma biomarkers, PFS and OS were analyzed by baseline levels and on-treatment changes for both cabozantinib and everolimus in the METEOR trial.

## Methods

### Study design

Details of the METEOR study design have been published [3, 4]. The study was registered at ClinicalTrials.gov (NCT01865747, 05/31/2013). The study adhered to the Good Clinical Practice guidelines, the Declaration of Helsinki, and all applicable local laws and regulatory requirements. The study protocol was approved by the institutional review board or ethics committee of all participating centers (the names of the institutional review boards or ethics committees are provided in the Supplement). All patients provided written informed consent.

Patients with advanced RCC with a clear cell component who had been previously treated with up to two prior VEGFR-tyrosine kinase inhibitors (TKIs) were randomized 1:1 to receive cabozantinib (60 mg daily) or everolimus (10 mg daily). Randomization was stratified by the number of prior VEGFR-TKIs and MSKCC risk group. The primary endpoint was PFS per RECIST 1.1 per independent review committee (IRC), and secondary endpoints were OS and ORR per RECIST 1.1 per IRC. PFS was defined as the time from randomization to radiographic progression or death from any cause. OS was defined as the time from randomization to death from any cause. The relationship of baseline and on-treatment plasma biomarkers with outcomes was an exploratory endpoint.

### Assessments and biomarker samples

Computed tomography (CT) or magnetic resonance imaging (MRI) scans of the chest, abdomen, and pelvis were performed at screening, every 8 weeks for the first 12 months, and every 12 weeks thereafter.

Blood (2 mL) for plasma samples was collected at baseline and at week 4 (week 5 day 1) in K2-EDTA Vacutainer tubes (BD) and processed into plasma within 30 min by spinning in a refrigerated centrifuge to separate the plasma. Plasma was aliquoted into cryovials and frozen before storing at − 70 °C. Plasma protein levels of CA9, HGF, MET, GAS6, AXL, VEGF, VEGFR2, and IL-8 were measured by ELISA or Luminex assay platforms (Assay Gate, Ijamsville, MD).

### Statistical analysis

Analyses included all patients from the METEOR trial database with available biomarker data. The data cutoff was May 22, 2015 for PFS, and October 31, 2015 for OS.

Baseline biomarkers were considered correlated if *p* < 0.05 with a Spearman correlation coefficient of ≥0.25 for pairwise interactions. Fold change at week 4 was defined as the biomarker level at week 4 divided by the baseline biomarker level; fold change above or below 1 represents an increase or decrease, respectively, in the biomarker. Baseline and week 4 measurements were paired for each patient to calculate fold change. On-treatment changes in biomarker levels were evaluated for significance (*p* < 0.05) using the paired t-test. Association of baseline biomarker levels with IMDC risk group was evaluated by ANOVA using a linear model to test for the difference between group means.

PFS and OS were analyzed using the log-rank test and Cox proportional hazards model. For analyses of baseline biomarkers, PFS and OS were analyzed by subgroups of high vs low biomarker levels dichotomized at the median for each treatment arm. PFS and OS were also analyzed with baseline biomarker levels as a continuous variable, with baseline biomarker levels normalized by log_2_-transformation in each treatment arm. Analyses of PFS and OS for cabozantinib versus everolimus by subgroups of baseline biomarker levels dichotomized at the median were also conducted. For on-treatment changes, PFS and OS were analyzed using the log_2_-transformation of the fold change at week 4 as a continuous variable. Biomarkers were considered prognostic for PFS and OS if *p* < 0.05 for the analyses. Biomarkers were identified as predictive if the *p*-value for the interaction between treatment and biomarker level (p_interaction_) was < 0.05. Univariate analyses were conducted initially, followed by multivariable analyses that included International Metastatic Renal Cell Carcinoma Database Consortium (IMDC) risk groups (favorable, intermediate, or poor) [14] as cofactors in the model to adjust for differences in baseline risk group status. Separate multivariable analyses that included all biomarkers (baseline and on-treatment changes) were also conducted to determine if biomarkers were independently prognostic; these were run sequentially with baseline biomarkers dichotomized at the median and also with biomarkers expressed as the continuous log_2_ of baseline levels.

The relationship of baseline and on-treatment plasma biomarkers with outcomes was an exploratory endpoint of the METEOR trial, and the analyses conducted here are retrospective. *P*-values were not adjusted for multiplicity as these are exploratory analyses.

## Results

### Patients and baseline plasma biomarker levels and on-treatment changes

A total of 330 patients were randomized to receive cabozantinib and 328 were randomized to receive everolimus. Demographics and baseline characteristics were generally balanced between treatment arms [3, 4]. Nineteen percent of patients were favorable risk, 64% were intermediate risk, and 16% were poor risk according to IMDC prognostic criteria. The majority of patients were male (75%) with a median age of 62 years. Plasma samples were available for the majority of patients (94% at baseline). Baseline characteristics for patients with biomarker data were similar to those for the randomized population, including for IMDC risk group, sites of metastases, prior therapy and prior nephrectomy (Supplementary Table 1).

Biomarkers evaluated in this study were CA9, HGF, MET, GAS6, AXL, VEGF, VEGFR2, and IL-8. Biomarker levels were measured from collected plasma samples at baseline and week 4 (Table 1). Baseline biomarker data were available for 316/330 patients in the cabozantinib arm and 305/328 patients in the everolimus arm, and fold change data at week 4 were available for 304/330 and 280/328 patients, respectively. Median baseline levels of each of the plasma biomarkers were similar when comparing the cabozantinib and everolimus arms (Table 1). Some of the biomarker levels were found to be correlated at baseline in pairwise analyses (correlation coefficient ≥ 0.25 and *p* < 0.05; Supplementary Figure 1); all biomarker pairs had significant but weak correlations; the highest correlation coefficients (~ 0.4) were observed between AXL and GAS6, VEGF and IL8, and MET and GAS6. An ANOVA was performed to assess the association between baseline biomarkers levels and IMDC risk groups. All biomarker levels were significantly different between IMDC risk groups except for CA9, with the largest variance observed for HGF and VEGF between IMDC groups (Supplementary Table 2). For all biomarkers, mean levels were highest in the poor risk group, and for most (HGF, MET, GAS6, AXL, VEGF, and IL-8) relative levels were poor>intermediate>favorable; for VEGFR2, levels were poor>favorable>intermediate.

**Table 1:** Biomarker levels at baseline and week 4

Plasma Biomarker	Cabozantinib ***N*** = 330				Everolimus ***N*** = 328			
	Baseline Level	Fold Change at week 4		***p***-value	Baseline Level	Fold Change at week 4		***p***-value
	Median	Median	Mean		Median	Median	Mean	
CA9	77.92	1.93	4.84	< 0.0001	87.49	1.15	2.60	< 0.0001
HGF	745.0	0.86	1.09	< 0.0001	721.1	1.09	1.51	0.002
MET	182.8	1.09	1.13	< 0.0001	178.0	0.98	1.00	0.05
GAS6	14,760	1.40	1.51	< 0.0001	14,570	1.12	1.11	< 0.0001
AXL	14,820	1.14	1.20	< 0.0001	14,690	0.92	0.94	< 0.0001
VEGF	11.55	2.57	6.74	< 0.0001	11.20	1.11	3.65	0.005
VEGFR2	4699	0.79	0.85	< 0.0001	4918	0.84	0.89	< 0.0001
IL-8	4.601	1.23	3.44	0.004	5.049	1.32	6.46	< 0.0001

All values in pg/mL except for MET which is in ng/mL

Plasma biomarker baseline and fold change data were available for 316 and 304 patients in the cabozantinib arm and 305 and 280 patients in the everolimus arm, respectively, with the exception of for IL-8 in the everolimus arm, for which 304 and 279 patients had available data, respectively

At week 4, all biomarkers had significant changes from baseline in both treatment arms (Table 1), with most biomarker levels increasing (fold change> 1). In the cabozantinib arm, mean biomarker levels of CA9, HGF, MET, GAS6, VEGF, and IL-8 increased, and mean levels of VEGFR2 decreased (fold change< 1). In the everolimus arm, mean levels of CA9, HGF, GAS6, VEGF, and IL-8 increased, and mean levels of VEGFR2 decreased. Biomarkers with the largest increases at week 4 were VEGF (mean fold change of 6.7 with cabozantinib and 3.6 with everolimus), CA9 (4.8 and 2.6), and IL-8 (3.4 and 6.5); all other biomarkers had mean fold increases of ≤1.5 in both treatment arms.

### Progression-free survival and overall survival by baseline biomarker levels

**Fig. 1:**
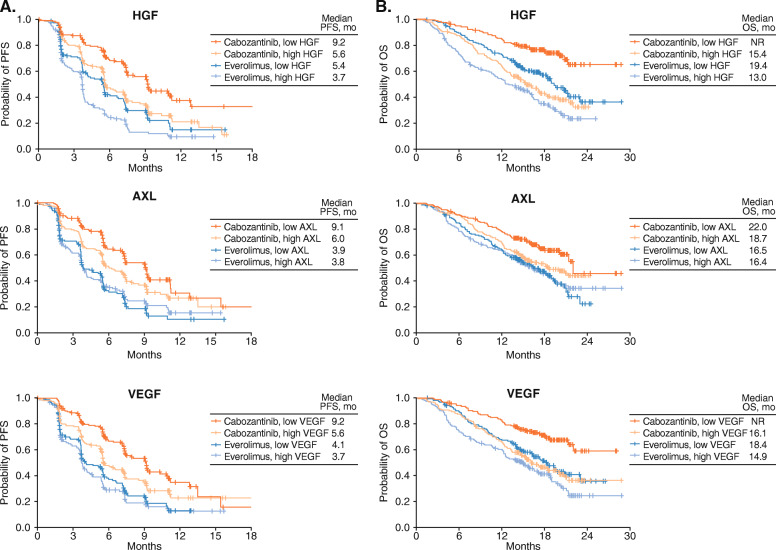
Kaplan-Meier plot of progression-free survival (**A**) and overall survival (**B**) by baseline biomarker levels dichotomized at the median

PFS and OS were analyzed by baseline biomarker levels (≥median vs < median) for each treatment arm. Results for PFS are shown in Table 2. Low vs high baseline levels of HGF, AXL, and VEGF were associated with longer PFS with cabozantinib (*p* < 0.05), and low vs high baseline levels of HGF and IL-8 were associated with longer PFS with everolimus. Kaplan-Meier plots of PFS by high and low biomarker levels are shown for HGF, AXL, and VEGF in Fig. 1.

**Table 2:** Progression-free survival within each treatment arm by baseline biomarker levels dichotomized at the median

Plasma Biomarker	Cabozantinib			Everolimus			P_**interaction**_
	Median PFS, mo		HR (95% CI)	Median PFS, mo		HR (95% CI)	
	≥median biomarker	<median biomarker		≥median biomarker	<median biomarker		
CA9	7.4	7.4	0.87 (0.65, 1.17)	3.9	3.8	0.89 (0.67, 1.17)	1.0
HGF	5.6	9.2	1.77 (1.30, 2.40)*	3.7	5.4	1.48 (1.12, 1.95)*	0.47
MET	7.4	7.4	1.00 (0.74, 1.35)	3.7	4.1	1.16 (0.88, 1.53)	0.45
GAS6	7.2	9.1	1.31 (0.97, 1.77)	3.7	3.9	1.07 (0.81, 1.40)	0.36
AXL	6.0	9.1	1.45 (1.07, 1.96)*	3.8	3.9	0.92 (0.70, 1.21)	0.02*
VEGF	5.6	9.2	1.57 (1.17, 2.12)*	3.7	4.1	1.15 (0.87, 1.51)	0.16
VEGFR2	7.4	7.4	0.89 (0.66, 1.19)	4.4	3.7	0.88 (0.67, 1.16)	0.94
IL-8	7.3	7.4	1.10 (0.82, 1.49)	3.7	5.3	1.40 (1.06, 1.85)*	0.21

Hazard ratios are for high (≥median) versus low (<median) biomarker levels

P-interaction was obtained from a separate model that included the interaction between treatment and biomarker level

* *p* < 0.05 for the analysis

Analyses of PFS for cabozantinib versus everolimus were also conducted by subgroups of high and low biomarker level. Cabozantinib was associated with prolonged PFS compared with everolimus for both high and low levels of all biomarkers analyzed with all HRs ≤0.65 (Supplementary Table 3). Low levels of AXL were predictive for an improved relative PFS benefit with cabozantinib compared with everolimus (Table 2).

Analysis of OS by baseline biomarkers (≥median vs < median) for each treatment arm is shown in Table 3. Low vs high levels of HGF, GAS6, AXL, VEGF, and IL-8 were associated with longer OS with cabozantinib, and low vs high levels of HGF, MET, GAS6, VEGF, and IL-8 were associated with longer OS with everolimus (*p* < 0.05). Kaplan-Meier plots of OS by high and low biomarker levels are shown for HGF, AXL, and VEGF in Fig. 1.

**Table 3:** Overall survival within each treatment arm by baseline biomarker levels dichotomized at the median

Plasma Biomarker	Overall Survival						P_**interaction**_
	Cabozantinib			Everolimus			
	Median OS, mo		HR (95% CI)	Median OS, mo		HR (95% CI)	
	≥median biomarker	<median biomarker		≥median biomarker	<median biomarker		
CA9	21.4	22.0	1.19 (0.85, 1.68)	15.4	16.5	1.20 (0.89, 1.62)	0.99
HGF	15.4	NR	2.79 (1.92, 4.05)*	13.0	19.4	1.78 (1.32, 2.42)*	0.08
MET	19.9	NR	1.29 (0.91, 1.81)	15.0	18.9	1.42 (1.05, 1.91)*	0.70
GAS6	17.2	NR	2.01 (1.41, 2.86)*	13.9	18.4	1.42 (1.05, 1.92)*	0.14
AXL	18.7	22.0	1.48 (1.05, 2.09)*	16.4	16.5	1.00 (0.74, 1.35)	0.11
VEGF	16.1	NR	2.16 (1.51, 3.08)*	14.9	18.4	1.43 (1.06, 1.94)*	0.09
VEGFR2	21.4	22.0	0.97 (0.69, 1.36)	16.5	16.4	0.90 (0.66, 1.21)	0.75
IL-8	17.2	NR	1.90 (1.34, 2.68)*	13.0	19.4	1.78 (1.31, 2.42)*	0.73

Hazard ratios are for high (≥median) versus low (<median) biomarker levels. P-interaction was obtained from a separate model that included the interaction between treatment and biomarker level

*NR* not reached

* *p* < 0.05 for the analysis

Analyses of OS for cabozantinib versus everolimus were also conducted by subgroups of low and high biomarker level. Cabozantinib was associated with prolonged OS compared with everolimus (HR < 1) for both low and high levels of all biomarkers analyzed with all HRs ≤0.78 (Supplementary Table 3). None of the biomarkers were predictive for a differential treatment effect at a significance level of 0.05; HGF, GAS6, AXL, and VEGF had the lowest p_interaction_ values (p_interaction_ < 0.15) (Table 3).

PFS and OS were also analyzed with the log_2_ of baseline biomarker levels as continuous variables. In general, the analyses gave similar results to those using dichotomized levels, with decreasing levels of some biomarkers associated with improvements in PFS or OS (Supplementary Table 4). In the continuous analyses, additional biomarkers identified as prognostic for improved PFS were decreased levels of MET with cabozantinib, and additional biomarkers prognostic for improved OS were decreased levels of MET with cabozantinib and decreased levels of AXL and CA9 with everolimus. No biomarkers were predictive in analyses with baseline biomarker levels expressed as continuous variables; AXL had the lowest p-interaction value for PFS and GAS6 had the lowest p-interaction for OS (p_interaction_ values < 0.15).

### Multivariable analyses of baseline biomarkers adjusting for IMDC risk group

Baseline biomarkers dichotomized at the median were further investigated by multivariable analyses including IMDC risk group as a covariate in the model; results are shown in Table 4. Each biomarker was adjusted with IMDC risk group in separate analyses. All biomarkers identified as prognostic for PFS in the univariate analyses were independently prognostic when adjusting for IMDC risk group; low vs high baseline levels of HGF, AXL, and VEGF remained prognostic for improved PFS with cabozantinib, and low vs high baseline levels of HGF and IL-8 remained prognostic for improved PFS with everolimus. Low levels of AXL remained predictive for an improved relative PFS benefit with cabozantinib compared with everolimus. In multivariable analyses of OS adjusting for IMDC risk group, low vs high baseline levels of HGF, GAS6, VEGF, and IL-8 were independently prognostic for OS with cabozantinib. For everolimus, HGF, GAS6, and IL-8 were independently prognostic for OS.

**Table 4:** Multivariable analyses of PFS and OS including IMDC risk group in each treatment arm by baseline biomarker level dichotomized at the median

Plasma Biomarker	Progression-Free Survival HR (95% CI)			Overall Survival HR (95% CI)		
	Cabozantinib	Everolimus	P_**interaction**_	Cabozantinib	Everolimus	P_**interaction**_
HGF	1.57 (1.14, 2.16)*	1.39 (1.05, 1.84)*	0.53	2.28 (1.55, 3.35)*	1.57 (1.16, 2.13)*	0.16
MET	0.96 (0.71, 1.29)	1.11 (0.84, 1.47)	0.57	1.24 (0.88, 1.74)	1.33 (0.98, 1.8)	0.80
GAS6	1.29 (0.96, 1.74)	1.05 (0.80, 1.39)	0.35	1.83 (1.28, 2.6)*	1.37 (1.01, 1.86)*	0.20
AXL	1.39 (1.03, 1.88)*	0.87 (0.65, 1.15)	0.013*	1.40 (0.99, 1.98)	0.98 (0.72, 1.32)	0.14
VEGF	1.41 (1.03, 1.94)*	1.07 (0.81, 1.42)	0.16	1.72 (1.19, 2.49)*	1.34 (0.98, 1.82)	0.29
IL-8	1.03 (0.76, 1.40)	1.33 (1.01, 1.76)*	0.24	1.77 (1.25, 2.50)*	1.67 (1.23, 2.27)*	0.76

Hazard ratios are for high (≥median) versus low (<median) biomarker levels. IMDC risk groups were included as cofactors in the multivariable analysis. Biomarkers were included in the multivariable analysis that had *p* < 0.1 in at least one of the univariate analyses for PFS or OS by treatment arm. P-interaction was obtained from a separate model that included the interaction between treatment and biomarker level

* *p* < 0.05 for the analysis

Baseline biomarkers expressed as continuous variables were also run in multivariable analyses including IMDC risk group as a covariate in the model (Supplementary Table 5). Decreasing levels of baseline HGF and AXL were independently prognostic for improved PFS with cabozantinib and decreasing levels of baseline HGF were independently prognostic for improved PFS with everolimus. For OS, decreasing baseline levels of HGF, MET, GAS6, VEGF, and IL-8 were independently prognostic for cabozantinib, and decreasing baseline levels of HGF, MET, GAS6, VEGF, and IL-8 were independently prognostic with everolimus. No biomarkers were predictive for an improved relative PFS or OS benefit for cabozantinib or everolimus at a significance level of 0.05. AXL had the lowest p_interaction_ values for PFS and GAS6 had the lowest p_interaction_ value for OS (p_interaction_ values < 0.15).

### PFS and OS analyzed by continuous on-treatment changes in biomarker levels

Univariate analyses of PFS and OS based on the log_2_-transformation of fold change at week 4 as a continuous variable are shown in Table 5. In analyses of PFS, an increase in HGF at week 4 was associated with improved PFS in the cabozantinib group. An increase in HGF was also predictive for relative improvement in PFS for cabozantinib compared with everolimus (p_interaction_ = 0.02).

**Table 5:** Univariate analyses of progression-free survival and overall survival based on continuous log_2_ fold change in biomarkers at week 4

Plasma Biomarker	Hazard Ratio (95% CI) for PFS		P_**interaction**_	Hazard Ratio (95% CI) for OS		P_**interaction**_
	Cabozantinib	Everolimus		Cabozantinib	Everolimus	
CA9	1.00 (0.92, 1.08)	1.04 (0.95, 1.14)	0.48	0.92 (0.84, 1.00)	0.93 (0.83, 1.03)	0.86
HGF	0.77 (0.63, 0.93)*	1.06 (0.88, 1.28)	0.02*	0. 80 (0.65, 0.98)*	1.00 (0.80, 1.26)	0.14
MET	1.16 (0.74, 1.83)	0.70 (0.41, 1.19)	0.13	1.02 (0.62, 1.67)	0.79 (0.43, 1.42)	0.52
GAS6	0.87 (0.60, 1.28)	0.88 (0.61, 1.29)	0.92	0.67 (0.44, 1.01)	1.05 (0.68, 1.60)	0.13
AXL	0.67 (0.41, 1.11)	0.88 (0.54, 1.44)	0.53	0.70 (0.41, 1.19)	0.86 (0.51, 1.47)	0.55
VEGF	0.95 (0.89, 1.01)	1.03 (0.95, 1.11)	0.13	0.90 (0.84, 0.97)*	0.92 (0.85, 1.01)	0.63
VEGFR2	1.05 (0.69, 1.60)	0.86 (0.54, 1.34)	0.52	1.06 (0.70, 1.61)	0.86 (0.53, 1.42)	0.48
IL-8	0.93 (0.85, 1.01)	1.00 (0.92, 1.09)	0.26	0.91 (0.83, 1.00)*	0.96 (0.88, 1.06)	0.37

P-interaction was obtained from a separate model that included the interaction between treatment and biomarker level

* *p* < 0.05 for the analysis

In analyses of OS, an increase in HGF, VEGF, or IL-8 at week 4 was associated with improved OS in the cabozantinib group (Table 5). For the everolimus group, none of the on-treatment changes in biomarker level were associated with OS. No biomarkers were predictive for a differential treatment effect; the lowest p_interaction_ values (p_interaction_ < 0.15) were for HGF and GAS6.

### Multivariable analyses of PFS and OS with multiple biomarkers

Biomarkers were further investigated by multivariable analyses including all biomarkers which had *p* < 0.10 in the univariate analyses. Multivariable analyses were run separately for baseline biomarkers dichotomized at the median (Table 6) and baseline biomarkers as continuous variables (Supplementary Table 6) with each analysis including on-treatment changes in biomarkers as covariates. For analyses including baseline biomarkers dichotomized at the median, low vs high levels of HGF were independently prognostic for improved PFS for both cabozantinib and everolimus. For OS, low vs high levels of HGF, GAS6, and VEGF were independently prognostic for OS with cabozantinib; no biomarkers were independently prognostic for OS with everolimus.

**Table 6:** Multivariable analyses of progression-free survival and overall survival in each treatment arm including baseline biomarkers dichotomized at the median and change in biomarkers at week 4 as covariates

Plasma Biomarker	HR (95% CI)	***P***_**value**_
**Progression-Free Survival (Cabozantinib)**		
HGF	1.46 (1.02, 2.08)	0.04*
VEGF	1.32 (0.95–1.83)	0.10
AXL	1.31 (0.95, 1.79)	0.10
∆HGF	0.87 (0.70, 1.07)	0.19
GAS6	1.08 (0.79, 1.49)	0.62
∆IL8	0.98 (0.90, 1.07)	0.68
**Overall Survival (Cabozantinib)**		
HGF	2.17 (1.41, 3.35)	< 0.001*
GAS6	1.62 (1.08–2.42)	0.02*
VEGF	1.62 (1.04, 2.53)	0.03*
AXL	1.19 (0.83, 1.72)	0.34
IL8	1.15 (0.76, 1.76)	0.51
∆CA9	0.97 (0.87–1.07)	0.52
∆VEGF	1.02 (0.93, 1.11)	0.74
∆HGF	0.98 (0.77, 1.23)	0.85
∆GAS6	0.98 (0.6, 1.59)	0.93
∆IL8	1 (0.89, 1.13)	0.94
**Progression-Free Survival (Everolimus)**		
HGF	1.37 (1.03, 1.82)	0.03*
IL8	1.29 (0.97, 1.71)	0.08
**Overall Survival (Everolimus)**		
MET	1.37 (0.99, 1.90)	0.06
HGF	1.38 (0.99, 1.93)	0.06
IL8	1.35 (0.95–1.91)	0.09
GAS6	1.20 (0.86, 1.67)	0.28
∆VEGF	0.96 (0.87, 1.06)	0.46
VEGF	1.07 (0.74, 1.55)	0.72

Biomarkers were included in the multivariable analysis if *p* < 0.10 in the univariate analyses. Hazard ratios are for high versus low biomarker levels. ∆ Indicates the covariate is change in the biomarker at week 4; all other covariates are baseline biomarkers dichotomized at the median

* *p* < 0.05 for the analysis

For analyses including baseline biomarkers expressed as continuous variables, decreasing levels of AXL were independently prognostic for improved PFS with cabozantinib and decreasing levels of HGF were prognostic for improved PFS with everolimus. For OS, decreasing levels of HGF and GAS6 were both independently prognostic for improved OS with cabozantinib and decreasing levels of HGF were independently prognostic for improved OS with everolimus.

No on-treatment changes in biomarkers were independently prognostic for PFS or OS in any of the multivariable analyses (Table 6 and Supplementary Table 6).

## Discussion

The METEOR trial showed that cabozantinib improved PFS, OS, and ORR compared with everolimus in patients with advanced RCC who received prior antiangiogenic therapy. In the current study, PFS and OS were analyzed based on eight plasma biomarkers to test for prognostic and predictive significance: CA9, HGF, MET, GAS6, AXL, VEGF, VEGFR2, and IL-8. These biomarkers were selected based on biological relevance to renal cell carcinoma, previous reports of prognostic significance, and the target profile of cabozantinib including both cabozantinib receptor targets (VEGFR2, MET, and AXL) and their ligands (VEGF, HGF, and GAS6). CA9 and IL-8 were selected based on previous reports of prognostic significance in RCC [15–17].

PFS and OS favored cabozantinib versus everolimus for both low and high baseline levels of all biomarkers analyzed (hazard ratios ≤0.78), suggesting a benefit with cabozantinib treatment irrespective of biomarker status. In univariate analyses of the cabozantinib arm based on high vs low baseline biomarker levels, low HGF, AXL, and VEGF were prognostic for improved PFS and low HGF, GAS6, AXL, VEGF, and IL-8 were prognostic for improved OS. In the everolimus arm, low HGF and IL-8 were prognostic for improved PFS and low HGF, MET, GAS6, VEGF, and IL-8 were prognostic for improved OS. Results with baseline biomarkers expressed as continuous variables were generally consistent.

Multivariable analyses were performed to determine if biomarkers were independently prognostic for outcome. In analyses adjusting for IMDC risk group, low HGF was independently prognostic for improved PFS and OS, and low GAS6 and IL-8 were independently prognostic for improved OS, for both cabozantinib and everolimus. Low VEGF was independently prognostic for improved OS for cabozantinib only. Baseline levels of all biomarkers were significantly different between IMDC risk groups except for CA9, suggesting an association of biomarker level with risk status, which has been previously reported for VEGF and MSKCC risk status [18]. Separate multivariable analyses for outcome including all baseline biomarkers were performed to test for the independence of baseline biomarkers with respect to each other. In these analyses low baseline HGF was independently prognostic for improved PFS for both cabozantinib and everolimus in most of the analyses, although it did not meet the threshold for prognostic significance with cabozantinib in analyses based on continuous biomarker level. Low baseline HGF and GAS6 were independently prognostic for improved OS with cabozantinib in all of the analyses.

Low HGF, VEGF, and IL-8 have all been implicated as prognostic biomarkers for improved PFS or OS in previous studies of RCC with targeted therapy, interferon-α, or placebo [17–20]. GAS6 and its cognate receptor AXL have not been extensively studied as biomarkers, but higher levels of expression of both GAS6 and AXL have been reported to be associated with worse survival [12]. That low levels of both HGF and GAS6 demonstrated prognostic significance with cabozantinib treatment across multiple analyses is relevant for several reasons. First, these biomarkers are ligands for two of the key targets inhibited by cabozantinib (MET and AXL), and these targets differentiate cabozantinib from other TKIs. In the current study, patients with low levels of HGF had a more favorable prognosis for PFS and OS in both treatment arms, although there was a numerically greater improvement for cabozantinib compared with everolimus in many of the analyses. The results presented here show that HGF and GAS6 have prognostic significance for cabozantinib monotherapy; however, we hypothesize that these biomarkers could also have prognostic significance for cabozantinib in combination with other agents including checkpoint inhibitors. The RCC treatment landscape is evolving, and cabozantinib has recently been approved in combination with nivolumab for patients with previously untreated advanced RCC [21]. Biomarker studies of cabozantinib plus nivolumab and other therapies should evaluate HGF and GAS6 levels to further assess their significance as prognostic and predictive biomarkers in RCC.

Pharmacodynamic changes for cabozantinib targets were consistent with previous reports with cabozantinib treatment, with all biomarkers increasing except for VEGFR2, which decreased in both arms [22, 23]. The largest on-treatment increases were observed for VEGF, CA9, and IL-8 in both treatment arms. Increases in VEGF and CA9 and decreases in VEGFR2 with cabozantinib treatment have been observed in triple negative breast cancer [22], and increases in VEGF, CA9, MET, and IL-8 and decreases in VEGFR2 with cabozantinib treatment have been observed in castration-resistant prostate cancer [23]. Increases in VEGF and decreases in VEGFR2 with other TKIs that inhibit VEGFR2, including sunitinib and sorafenib, have also been observed in RCC [17, 20, 24]. Although on-treatment changes in some biomarkers (HGF, VEGF, IL-8) appeared prognostic for improved PFS or OS with cabozantinib in univariate analyses, none of these were independently prognostic in multivariable analyses.

The METEOR trial was not powered to evaluate outcomes based on biomarkers, which was an exploratory endpoint. Nonetheless, a large number of patients in this phase 3 trial had data available for the analyses. The current study identified several potential prognostic baseline biomarkers; however, the lack of a placebo arm limits the ability to separate the prognostic effect from the treatment effect. The analyses presented here focused on plasma biomarkers because of the relative ease of obtaining contemporaneous samples from patients. Tumor MET status from available archival or recently biopsied tumor tissue has also been analyzed in the METEOR trial and was not found to be predictive of benefit with cabozantinib [25].

No plasma biomarkers were found to be consistently predictive for an improved benefit in the analyses; however, low baseline levels of AXL were predictive for improved PFS with cabozantinib in some of the analyses. Evaluation of interaction terms for predictive biomarkers requires anywhere from 4 to 16 times the sample size needed for testing main effects, and one approach to address this is to raise the cutoff for the *p*-value when considering interactions [26]. Clear predictive biomarkers for treatment of RCC have not been reported [27], and some studies have taken the approach of using multiple biomarkers to calculate a composite biomarker score for benefit, including for everolimus versus sunitinib [19] and more recently, for lenvatinib plus everolimus versus everolimus alone [28]. Additional studies are needed to identify predictive biomarkers in advanced RCC.

## Conclusions

In the METEOR trial, multiple baseline plasma biomarkers were prognostic for PFS or OS with cabozantinib or everolimus treatment. In particular, low baseline levels of HGF and GAS6, cognate ligands for MET and AXL, were prognostic for improved PFS or OS with cabozantinib treatment, supporting further studies of these biomarkers in patients with advanced RCC, where several TKI-immuno-oncology combinations, including cabozantinib and nivolumab, have demonstrated clinical benefit.

## Supplementary Information

**Additional file 1.**

## Data Availability

The datasets used and/or analyzed during the current study are available from the corresponding author or sponsor author on reasonable request.

## Abbreviations

CT: Computed tomography
IMDC: International Metastatic Renal Cell Carcinoma Database Consortium
IRC: Independent review committee
MRI: Magnetic resonance imaging
ORR: Objective response rate
OS: Overall survival
PFS: Progression-free survival
RCC: Renal cell carcinoma
TKI: Tyrosine kinase inhibitor
