# Correction to: Outcomes based on plasma biomarkers in METEOR, a randomized phase 3 trial of cabozantinib vs everolimus in advanced renal cell carcinoma

**DOI:** 10.1186/s12885-021-08693-9

**Published:** 2021-09-15

**Authors:** Thomas Powles, Toni K. Choueiri, Robert J. Motzer, Eric Jonasch, Sumanta Pal, Nizar M. Tannir, Sabina Signoretti, Rajesh Kaldate, Christian Scheffold, Evelyn Wang, Dana T. Aftab, Bernard Escudier, Daniel J. George

**Affiliations:** 1 Barts Cancer Institute, Queen Mary University of London, London, UK; 2 Dana-Farber Cancer Center, Boston, MA USA; 3 Memorial Sloan Kettering Cancer Center, New York, NY USA; 4 University of Texas MD Anderson Cancer Center, Houston, TX USA; 5 City of Hope National Medical Center, Duarte, CA USA; 6 Brigham and Women’s Hospital, Boston, MA USA; 7 Exelixis, Inc, Alameda, CA USA; 8 Gustave-Roussy, Villejuif, France; 9 Duke Cancer Institute, Durham, NC USA

**Correction to: BMC Cancer 21, 904 (2021)**

**https://doi.org/10.1186/s12885-021-08630-w**

Following publication of the original article [1], it was noticed that uncorrected page proofs were mistakenly published. The publishers apologise for this error. The original article [1] has been corrected.

Below is a table of corrections which have been implemented in the original article.

**Table:** 

**Section**	**Originally published text**	**Corrected text**
Article note	Rajesh Kaldate^7^ & Evelyn Wang^7^	Rajesh Kaldate^7†^ & Evelyn Wang^7†^ ^†^Affiliation at the time of the study.
Abstract	Trial registration: ClinicalTrials.gov NCT01865747 (registered on 05/31/2013).	Trial registration: ClinicalTrials.gov NCT01865747 (registered on 05/31/2013). https://clinicaltrials.gov/ct2/show/NCT01865747
Table 1 note	Plasma biomarker baseline and fold change data were available for 316 and 304 patients in the cabozantinib arm and 305 and 280 patients in the everolimus arm, respectively, with the exception of for IL-8 in the everolimus arm, for which 304 and 279 patients had available data, respectively	Plasma biomarker baseline and fold change data were available for 316 and 304 patients in the cabozantinib arm and 305 and 280 patients in the everolimus arm, respectively, with the exception of IL-8 in the everolimus arm, for which 304 and 279 patients had available data, respectively
Figure 1	Footnote is missing	High levels and low levels are defined by ≥median and < median, respectively. NR, not reached.
Row 2	1.32 (0.95–1.83)	1.32 (0.95, 1.83)
Row 6	ΔIL8	ΔIL-8
Table 6 Row 11	IL8	IL-8
Table 6 Row 12	0.97 (0.87–1.07)	0.97 (0.87, 1.07)
Table 6 Row 16	ΔIL8	ΔIL-8
Table 6 Row 18	IL8	IL-8
Table 6 Row 21	IL8 / 1.35 (0.95–1.9)	IL-8 / 1.35 (0.95, 1.9)
Table 6 note	Biomarkers were included in the multivariable analysis if *p* < 0.10 in the univariate analyses. Hazard ratios are for high versus low biomarker levels. Δ Indicates the covariate is change in the biomarker at week 4; all other covariates are baseline biomarkers dichotomized at the median * *p* < 0.05 for the analysis	Biomarkers were included in the multivariable analysis if *p* < 0.10 in the univariate analyses. Hazard ratios are for high versus low biomarker levels. Δ indicates the covariate is change in the biomarker at week 4; all other covariates are baseline biomarkers dichotomized at the median * *p* < 0.05 for the analysis
Competing interests	Dr. Powles has received honoraria from Astellas Pharma, AstraZeneca, Bristol-Myers Squibb, Eisai, Exelixis, Incyte, Ipsen, Johnson & Johnson, Merck, Merck, Merck Serono, MSD, Novartis, Pfizer, Roche, and Seattle Genetics; has a consulting or advisory role with Astellas Pharma, AstraZeneca, Bristol-Myers Squibb, Eisai, Exelixis, Incyte, Ipsen, Johnson & Johnson, Merck, Merck Serono, MSD, Novartis, Pfizer, Roche, and Seattle Genetics; has received research funding from Astellas Pharma, AstraZeneca, Bristol-Myers Squibb, Eisai, Exelixis, Ipsen, Johnson & Johnson, Merck, Merck Serono, MSD, Novartis, Pfizer, Roche, and Seattle Genetics; and has received travel/accommodation/other expenses from AstraZeneca, Ipsen, MSD, Pfizer, and Roche.	Dr. Powles has received honoraria from Astellas Pharma, AstraZeneca, Bristol-Myers Squibb, Eisai, Exelixis, Incyte, Ipsen, Johnson & Johnson, Merck, Merck Serono, MSD, Novartis, Pfizer, Roche, and Seattle Genetics; has a consulting or advisory role with Astellas Pharma, AstraZeneca, Bristol-Myers Squibb, Eisai, Exelixis, Incyte, Ipsen, Johnson & Johnson, Merck, Merck Serono, MSD, Novartis, Pfizer, Roche, and Seattle Genetics; has received research funding from Astellas Pharma, AstraZeneca, Bristol-Myers Squibb, Eisai, Exelixis, Ipsen, Johnson & Johnson, Merck, Merck Serono, MSD, Novartis, Pfizer, Roche, and Seattle Genetics; and has received travel/accommodation/other expenses from AstraZeneca, Ipsen, MSD, Pfizer, and Roche.
Competing interests	Dr. Wang is employed by Exelixis; and has stock/ownership interests with Exelixis.	Dr. Wang is a former employee of Exelixis; has stock/ownership interests with Exelixis.
Additional file 1	‘Track changed’ version was published	Clean version is published this correction article and the original article has been udpated

## Supplementary Information

**Additional file 1.**
