# Supplementary material for: Correction to: Outcomes based on plasma biomarkers in METEOR, a randomized phase 3 trial of cabozantinib vs everolimus in advanced renal cell carcinoma
Source: BMC Cancer. 2021 Sep 15;21:1023. doi: 10.1186/s12885-021-08693-9 (PMC8442414; doi:10.1186/s12885-021-08693-9)
Supplement: Supplementary file 1 — Additional file 1. [file 12885_2021_8693_MOESM1_ESM.doc]

**SUPPLEMENT**

**Outcomes based on plasma biomarkers in METEOR, a randomized phase 3 trial of cabozantinib vs everolimus in advanced renal cell carcinoma**

Thomas Powles, Toni K Choueiri, Robert J Motzer, Eric Jonasch, Sumanta Pal, Nizar M Tannir, Sabina Signoretti, Rajesh Kaldate, Christian Scheffold, Evelyn Wang, Dana T Aftab, Bernard Escudier, Daniel J George

**List of ethics committee/independent review board approving the METEOR** study protocol

| Study Site | Name of Ethics Committee/Independent Review Board |
| --- | --- |
| **Australia** |  |
| Port Macquarie Base Hospital | St Vincent’s Hospital Human Research Ethics Committee |
| Princess Alexandra Hospital | St Vincent’s Hospital Human Research Ethics Committee |
| Eastern Health,  Box Hill Hospital | St Vincent’s Hospital Human Research Ethics Committee |
| St George Private Hospital | Bellberry Human Research Ethics Committee |
| St Vincent’s Hospital | St Vincent’s Hospital Human Research Ethics Committee |
| Westmead Hospital | St Vincent’s Hospital Human Research Ethics Committee |
| Prince of Wales Hospital | St Vincent’s Hospital Human Research Ethics Committee |
| Icon Cancer Care | Bellberry Human Research Ethics Committee |
| Sydney Adventist Hospital | Bellberry Human Research Ethics Committee |
| Royal Hobart Hospital | Tasmania Health & Medical Human Research Ethic Committee |
| Moorabin Hospital | St Vincent’s Hospital Human Research Ethics Committee |
| Border Medical Oncology | Bellberry Human Research Ethics Committee |
| Royal Adelaide Hospital | Royal Adelaide Hospital Research Ethics Committee |
| **Austria** |  |
| Kaiser-Franz-Josef Spital | Ethikkommission der Medizinischen, Universität Wien |
| Krankenhaus der Barmherzigen Schwestern Linz | Ethikkommission der Medizinischen, Universität Wien |
| AKH Univ Klink für Innere Medizin 1- Abt Klin Onkologie | Ethikkommission der Medizinischen, Universität Wien |
| **Belgium** |  |
| AZ Klina | UZ Leuven, Commissie voor Medische Ethiek |
| Institut Jules Bordet | UZ Leuven, Commissie voor Medische Ethiek |
| UZ Leuven, Campus Gasthuisberg | UZ Leuven, Commissie voor Medische Ethiek |
| Imelda Hospital | UZ Leuven, Commissie voor Medische Ethiek |
| **Canada** |  |
| Cross Cancer Institute | Health Research Ethics Board of Alberta (HREBA - Cancer Committee) |
| Sunnybrook Research Institute/Sunnybrook Health Sciences Ctr | Ontario Cancer Research Ethics Board (OCREB) |
| CancerCare Manitoba | University of Manitoba, Biomedical Research Ethics Board |
| Tom Baker Cancer Centre, Foot Hills Medical Centre | Alberta Cancer Research Ethics Committee |
| Juravinski Cancer Centre | Ontario Cancer Research Ethics Board (OCREB) |
| University Health Network, Princess Margaret Cancer Center | Ontario Cancer Research Ethics Board |
| British Columbia Cancer Agency - Vancouver Centre | UBC BCCA Research Ethics Board |
| Hôpital Notre-Dame du Centre Hospitalier de l’Université de Mtl | Comité d’évaluation scientifique et d’éthique du CHUM |
| London Health Sciences Centre | Ontario Cancer Research Ethics Board |
| QEII Health Sciences Centre | Capital Health Research Ethics Board |
| Lakeridge Health Oshawa | Lakeridge Health Research Ethics Board |
| **Chile** |  |
| Centro Internacional de Estudios Clinicos | Comité de Ética de la Investigacion del Servicio de Salud |
| Fundación Arturo López Pérez | Comité de Ética Cientifico del Servicio de Salud Metropolitano Oriente |
| **Czech Republic** |  |
| Fakultni nemocnice u sv. Anny v Brné | Etická komise Fakultni nemocnice u sv. Anny v Brné |
| Fakultni nemocnice Olomouc | Etická komise Fakultni nemocnice Olomouc |
| **Denmark** |  |
| Aarhus Universitets Hospital | De Videnskabsetiske Komitéer for Region Midtjylland |
| Herlev Universitets Hospital Onkologisk afdeling R | De Videnskabsetiske Komitéer for Region Midtjylland |
| Odense Universitetshospital Onkologisk afdeling | De Videnskabsetiske Komitéer for Region Midtjylland |
| **Finland** |  |
| Helsinki University Central Hospital | TUKIJA |
| **France** |  |
| JUCT-O - Institut Claudius Regaud - Oncologie Médicale | Comité de Protection des Personnes Ile de France III, Hôpital Tarnier |
| Institut Gustave Roussy - Médecine Oncologique | Comité de Protection des Personnes Ile de France III, Hôpital Tarnier |
| Institut Paoli Calmettes - Département d’oncologie médicale | Comité de Protection des Personnes Ile de France III, Hôpital Tarnier |
| Hôpital Saint André - CHU de Bordeaux - Oncologie Médicale | Comité de Protection des Personnes Ile de France III, Hôpital Tarnier |
| Centre Eugéne Marquis - Service d’Oncologie Médicale | Comité de Protection des Personnes Ile de France III, Hôpital Tarnier |
| Centre Léon Berard - Département d’oncologie médicale | Comité de Protection des Personnes Ile de France III, Hôpital Tarnier |
| Hôpital Européen Georges Pomidou - Oncologie Médicale | Comité de Protection des Personnes Ile de France III, Hôpital Tarnier |
| Centre René Gauducheau - Service d’Oncologie Médicale | Comité de Protection des Personnes Ile de France III, Hôpital Tarnier |
| Centre Régional de Lutte contre le cancer - Francois Baclesse | Comité de Protection des Personnes Ile de France III, Hôpital Tarnier |
| CHRU Besançon - Hôpital Jean Minjoz | Comité de Protection des Personnes Ile de France III, Hôpital Tarnier |
| Clinique Victor Hugo - Centre Jean Bernard | Comité de Protection des Personnes Ile de France III, Hôpital Tarnier |
| **Germany** |  |
| Klinikum der Johann Wolfgang Goethe-Universität | Ethikkommission der Medizinischen Hochule Hannover |
| Onkodok GmbH | Ethikkommission der Medizinischen Hochule Hannover |
| Nationales Centrum für Tumorerkrankungen | Ethikkommission der Medizinischen Hochule Hannover |
| Medizinische Hochschule Hannover | Ethikkommission der Medizinischen Hochule Hannover |
| Universitätsklinikum der RWTH Aachen | Ethikkommission der Medizinischen Hochule Hannover |
| Urologische Klinik und Poliklinik der TU München | Ethikkommission der Medizinischen Hochule Hannover |
| Universitätsklinikum Hamburg-Eppendorf | Ethikkommission der Medizinischen Hochule Hannover |
| Universitätsklinikum Ulm | Ethikkommission der Medizinischen Hochule Hannover |
| Universitätsklinikum Freiburg | Ethikkommission der Medizinischen Hochule Hannover |
| LMU Klinikum der Universität München | Ethikkommission der Medizinischen Hochule Hannover |
| Universitätsklinikum Carl Gustav Carus an der TU Dresden | Ethikkommission der Medizinischen Hochule Hannover |
| **Hungary** |  |
| Jász-Nagykin-Szolnok Megyei Hetényi Géza Kórház-Rendelöintézet | Egészégügyi Tudományos Tanács Klinikal Farmakológiai Etikai Bizottság  Gyógyszerészeti és Egészégügyi Minöség- és Szervezetfejlesztési Intézet Országos Gyógyszerészeti Intézet Föigazgatóság |
| Orszáagos Onkológiai Intézet | Egészégügyi Tudományos Tanács Klinikal Farmakológiai Etikai Bizottság  Gyógyszerészeti és Egészégügyi Minöség- és Szervezetfejlesztési Intézet Országos Gyógyszerészeti Intézet Föigazgatóság |
| **Ireland** |  |
| Institute For Cancer Research | St James’s Hospital/Adelaide Meath & National Childrens Hospital Research Ethics Committee |
| Department of Medical Oncology | NRES Committee London – Brent |
| **Italy** |  |
| IRCCS Azienda Ospedaliera Universitaría San Martino – IST Istituto Nazionale per la Ricerca sul Cancro | Comitato Etico Refione Liguria - Sezione 2 c/o IRCCS Azienda Ospedaliera Iniversitaria San Martino  Comitato Etico Lazio 1 |
| Azienda USL8 - Presidio Ospedaliero San Donato | Comitato Etico Sperimentazione Clinica Medicinali della AUSL 8 di Arezzo  Comitato Etico Azienda Ospedaliera San Camillo Forlanini  Comitato Etico Lazio 1  Comitato Etico di Area Vasta Sud-Est |
| Azienda Unità Sanitaria Locale di Ravenna – Ospedale di Faenza | Comitato Etico di Area Vasta Romagna (CEAV) e Istituto Scientifico Romagnolo per lo Studio e la Cura dei Tumori (I.R.S.T)  Comitato Etico Lazio 1  Comitato Etico Azienda Ospedaliera San Camillo Forlanini |
| Instituto Scientifico Romagnolo per lo studio e la Cura dei Tumori (I.R.S.T.) | Comitato Etico di Area Vasta Romagna (CEAV) e Istituto Scientifico Romagnolo per lo Studio e la Cura dei Tumori (I.R.S.T)  Comitato Etico Lazio 1 |
| Azienda Ospedaliera “Istituti Ospitalieri” di Cremona | Comitato Etico Area Cremona Mantova Lodi  Comitato Etico Lazio 1 |
| Struttura Complessa di Oncologia -Azienda Ospedaliera S Maria di Terni | Comitato Etico Azienda Ospedaliera San Camillo Forlanini  Comitato Etico delle Aziende Sanitarie delľUmbria di Perugia  Comitato Etico Lazio 1 |
| Dipartimento Integrato di Oncologia, Ematologia e Patologie dell’apparato respiratorio | Segreteria Scientifica del Comitato Etico Provinciale di Modena  Comitato Etico Lazio 1 |
| Universitá Campus Bio-Medico di Roma (UCBM) - Poloclinico Universitario | Comitato Etico dell’Universitá Campus Bio-Medico di Roma  Comitato Etico Azienda Ospedaliera Sant’Andrea  Comitato Etico Lazio 1  Comitato Etico Azienda Ospedaliera San Camillo Forlanini  Comitato Etico del’Universitá “Sapienza” (Policlinico Umberto I - A.O.S. Andrea) |
| Azienda Ospedaliera San Camillo Forlanini | Comitato Etico Lazio 1 |
| **Korea, Republic of** |  |
| Seoul National University Hospital | Seoul National University Hospital IRB  Seoul National University Hospital Institutional Review Board |
| Department of oncology, University of Ulsan College of Medicine | Asan Medical Center Institutional Review Board |
| Samsung Medical Centre | Samsung Medical Center Institutional Review Board |
| Yonsei University Health System, Severance Hospital | Yonsei University Health System, Severance Hospital Institutional Review Board |
| **Netherlands** |  |
| MUMC | METC azM/UM |
| Leids Universitair Medisch Centrum | METC azM/UM |
| The Netherlands Cancer Institute | Academisch Ziekenhuis Maastricht METC |
| **Poland** |  |
| Wojewodzkie Centrum Onkologii | Komisja Bioetyczna Wojskowego Instytutu Medycznego |
| Wojskowy Instytut Medyczny, Klinika Onkologii | Komisja Bioetyczna Wojskowego Instytutu Medycznego |
| Szpital Kliniczny Przenienienia Panskiegio | Komisja Bioetyczna Wojskowego Instytutu Medycznego |
| Bialostockie Centrum Onkologii | Komisja Bioetyczna Wojskowego Instytutu Medycznego |
| **Portugal** |  |
| Centro Hospitalar Lisboa Norte - Hospital de Santa Maria | CEIC - Comissão de Ética para a Investigação Clinica |
| Hospital da Luz-Serviço de Oncologia Médica | CEIC - Comissão de Ética para a Investigação Clinica |
| Instituto Português de Oncologia do Porto Francisco Gentil, EPE | CEIC - Comissão de Ética para a Investigação Clinica |
| **Russian Federation** |  |
| Yaroslavl Region Budget Institution of Healthcare «Regional Clinical Oncology Hospital», | Russian Federation of Healthcare, Department for Government Regulation of Medicines Circulation, Ethics Council  Independent Interdisciplinary Committee of clinical trials ethical expertise |
| Budget Healthcare Institution of Omsk region “Clinical Oncology Dispensary” | Russian Federation of Healthcare, Department for Government Regulation of Medicines Circulation, Ethics Council  Local IEC within Budget Healthcare Institution of Omsk region “Clinical Oncology Dispensary” |
| Federal State Budget Institution “Russian Oncology Research Center n.a. N.N. Blokhin” of Russian Academy of Medical Sciences | Russian Federation of Healthcare, Department for Government Regulation of Medicines Circulation, Ethics Council  Local IEC within “Russian Oncology Research Center n.a. N.N. Blokhin” of Russian Academy of Medical Sciences |
| **Slovakia** |  |
| Urologicke oddelenie, Fakultna nemocnica s poliklinikou Zilina | Local Ethics Committee: Eticka komisia Fakultnej nemocnica s poliklinikou Zilina  Central Ethics Committee: Urad Presovskeho samospravneho kraja - Eticka komisia |
| UROCENTRUM Milab, s.r.o | Central Ethics Committee: Urad Presovskeho samospravneho kraja - Eticka komisia |
| **Spain** |  |
| Hospital Universitario 12 de Octubre. Servicio de Oncologia | CEIC-Parc de Salut MAR |
| Hospital Clinico Universitario de Valencia | CEIC-Parc de Salut MAR |
| Hospital Universitario Virgen del Rocio, Servicio de Oncologia | CEIC-Parc de Salut MAR |
| Hospital Universitario Central de Astunias. Medical Oncology | CEIC-Parc de Salut MAR |
| Instituto Catalán de Oncologia (ICO L’Hospitalet). Oncologia | CEIC-Parc de Salut MAR |
| Hospital Universitario Madrid Sanchinarro | CEIC-Parc de Salut MAR |
| Hospital Universitario Ramón y Cajal. Oncologia Médica | CEIC-Parc de Salut MAR |
| Hopital de la Santa Creu y Sant Pau. Oncologia Médica | CEIC-Parc de Salut MAR |
| Hospital del Mar. Oncologia Médica | CEIC-Parc de Salut MAR |
| Clinica Universidad de Navarra. Servicio de Oncologia | CEIC-Parc de Salut MAR |
| Hospital Clinico Universitario Virgen de la Victoria | CEIC-Parc de Salut MAR |
| Hospital Universitari Vall d’Hebron. Medical Oncology | CEIC-Parc de Salut MAR |
| **Sweden** |  |
| Dep. Of Oncology, University Hospital | Regionala Etikprövingsnämnden i Stockholm |
| Dep. Of Oncology, Karolinska University Hospital | Regionala Etikprövingsnämnden i Stockholm |
| Dep. Of Oncology, Norrlands University Hospital | Regionala Etikprövingsnämnden i Stockholm |
| **Taiwan, Province of China** |  |
| Taipei Veterans General Hospital | Institutional Review Board, Taipei Veterans General Hospital |
| National Taiwan University Hospital | Research Ethics Committee A, National Taiwan University Hospital |
| Taichung Veterans General Hospital | The Institutional Review Board of Taichung Veterans General Hospital |
| **Turkey** |  |
| TC SB Marmara Uni Pendik Egt&Ars Hst Tibbi Onkoloji Bolum | Ege Universitesi Tip Fakultesi Klinik Arastirmalar Etik Kurulu |
| Ege Universitesi Tip Fakultesi Tulay Aktas Onkoloji Hastanesi | Ege Universitesi Tip Fakultesi Klinik Arastirmalar Etik Kurulu |
| Gaziantep Uni. Tip Fakultesi Onkoloji Hastanesi | Ege Universitesi Tip Fakultesi Klinik Arastirmalar Etik Kurulu |
| Gazi Universitesi Tip Fakultesi Hastanesi, Ic Hastaliklari ABD | Ege Universitesi Tip Fakultesi Klinik Arastirmalar Etik Kurulu |
| **United Kingdom** |  |
| Sarah Cannon Research Institute | NRES Committee London – Brent |
| Beaston West of Scotland Cancer Centre | NRES Committee London – Brent |
| Dept. Medical Oncology Christie Hospital NHS Foundation Trust | NRES Committee London – Brent |
| Clatterbridge Cancer Centre NHS Foundation Trust | NRES Committee London – Brent |
| The Royal Marsden NHS Foundation Trust,  Royal Marsden Hospital | NRES Committee London – Brent |
| The Royal Marsden NHS Foundation Trust,  Royal Marsden Hospital | NRES Committee London – Brent |
| Aberdeen Royal Infirmary | NRES Committee London – Brent |
| Edinburgh Cancer Centre, Western General Hospital | NRES Committee London – Brent |
| Mount Vernon Cancer Centre, Mount Vernon Hospital | NRES Committee London – Brent |
| University Hospitals Birmingham NHS Foundation Trust, Queen Elizabeth Hospital | NRES Committee London – Brent |
| Centre for Medical Oncology, St Bartholomew’s Hospital | NRES Committee London – Brent |
| **United States of America** |  |
| Huntsman Cancer Institute/University of Utah | University of Utah Institutional Review Board |
| UPMC Cancer Pavillion | Western Institutional Review Board |
| Dana-Faber Cancer Institute | Dana-Faber Cancer Institute IRB |
| Mayo Clinic | Mayo Clinic Institutional Review Board |
| **Texas Breast Specialists-Houston Memorial City** | US Oncology, Inc., IRB |
| Medical University of South Carolina, Hollings Cancer Center | Western Institutional Review Board |
| Cedars-Sinai Medical Center | Office of Research Compliance and Quality Improvement |
| H. Lee Moffitt Cancer Center & Research Institute, Inc | Liberty IRB |
| Duke University Medical Center | Duke University Health System Institutional Review Board |
| Fox Chase Cancer Center | Institutional Review Board |
| Sarah Cannon Research Institute | Western Institutional Review Board |
| The Sidney Kimmel Comprehensive Cancer Center at John Hopkins | John Hopkins Medicine Institutional Review Board |
| University of Tennessee Medical Center | Western Institutional Review Board |
| Mayo Clinic | Mayo Clinic Institutional Review Board |
| University of Maryland, Greenebaum Cancer Center | Human Research Protections Office (HRPO) |
| Texas Oncology-Baylor Charles A. Sammons Cancer Center | US Oncology. Inc, IRB |
| UCLA Medical Plaza | UCLA Office of the Human Research Protection Program |
| Yale University School of Medicine | Yale University Human Investigation Committee |
| Boca raton Regional Hospital Lynn Cancer Institute | Western Institutional Review Board |
| Kaiser Permanente Medical Center* | Kaiser Foundation Research Institute, Kaiser Permanente Northern California (KPNC) Institutional Review Board (IRB) |
| Northwestern University Feinberg School of Medicine | Northwestern University Institutional Review Board |
| Sylvester Comprehensive Cancer Center/UMHC | University of Miami Institutional Review Board |
| Memorial Sloan Kettering Cancer Center | Memorial Sloan Kettering Cancer Center Institutional Review Board (IRB) |
| Martha Morehouse Medical Plaza | Western Institutional Review Board |
| City of Hope | Western Institutional Review Board |
| UC San Diego Moores Cancer Center | Western Institutional Review Board |
| University of Michigan Medical Center | University of Michigan Medical School Institutional Review Board (IRBMED) |
| Texas Oncology - Fort Worth | US Oncology, Inc, IRB |
| Cleveland Clinic Foundation | Cleveland Clinic Institutional Review Board |
| Washington University School of Medicine | Washington University School of Medicine, Human Research Protection Office (IRB) |
| Oregon Health and Science University | Oregon Health and Science University Research Integrity Office |
| Las Vegas Medical Clinic | US Oncology, Inc, IRB |
| Cancer Therapy & Research Center at The University of Texas Health Science Center at San Antonio; Institute for Drug Development | The University of Texas Health Science Center at San Antonio Institutional Review Board |
| Albany Medical Center | US Oncology, Inc., IRB |
| The University of Arizona Cancer Center | Western Institutional Review Board (WIRB) |
| UAB Comprehensive Cancer Center | Western Institutional Review Board |
| University of Chicago Medical Center | University of Chicago Institutional Review Board |
| The University of Texas MD Anderson Cancer Center-Department of Genitourinary Medical Oncology | The University of Texas MD Anderson Cancer Center - Institutional Review Board |
| Baptist Hospital of Miami | US Oncology, Inc., IRB |
| Seattle Cancer Care Alliance | Western Institutional Review Board (WIRB) |
| University of Iowa Hospitals and Clinics | Western Institutional Review Board |
| Karmanos Cancer Institute | Western Institutional Review Board  Washington State University (Institutional Review Board) |
| University of Kansas Cancer Center and Medical Pavilion | Human Subjects Committee, University of Kansas Medical Center |
| Banner MD Anderson Cancer Center | Banner Health Institutional Review Board - Oncology Panel |
| Texas Oncology-South Austin | US Oncology, Inc, IRB |

**Supplementary Tables and Figures**

**Supplementary Figure 1. Correlations between baseline biomarkers**


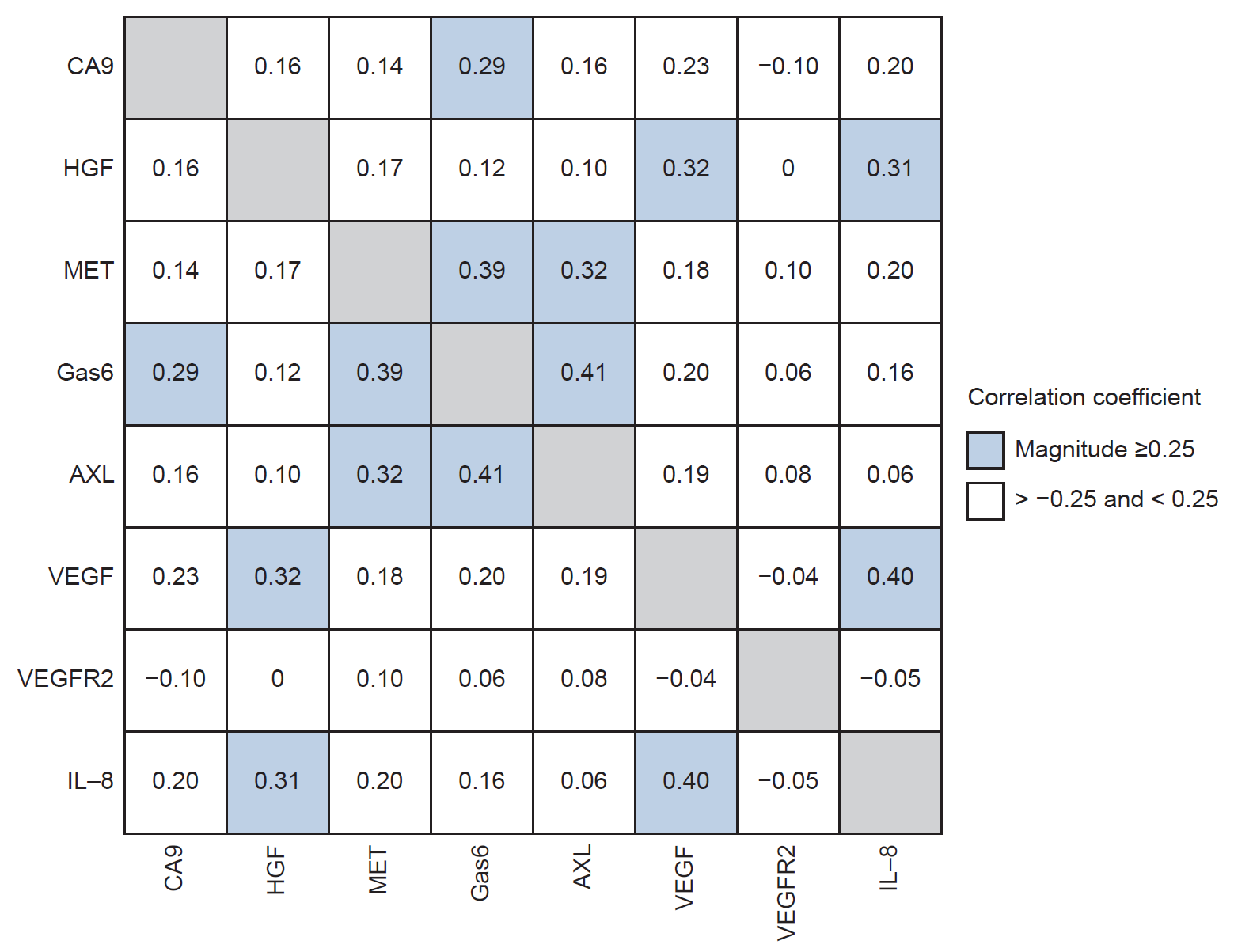


Spearman correlation coefficients are shown for baseline biomarker pairs. All pairwise correlations are significant (p<0.05). Biomarker pairs were considered correlated if p<0.05 with a correlation coefficient of magnitude ≥0.25 (colored in blue).

**Supplementary Table 1. Demographics and baseline characteristics**

| **Characteristic** | **All Randomized Patients** | | **Patients with Biomarker data** | |
| --- | --- | --- | --- | --- |
|  | **Cabozantinib N=330** | **Everolimus N=328** | **Cabozantinib N=316** | **Everolimus N=305** |
| Age, median (range), years | 63 (32, 86) | 62 (31, 84) | 62 (32, 86) | 62 (31, 84) |
| Male, n (%) | 253 (77) | 241 (73) | 243 (77) | 225 (74) |
| ECOG performance status, n (%) |  |  |  |  |
| 0 | 226 (68) | 216 (66) | 216 (68) | 197 (65) |
| 1 | 104 (32) | 112 (34) | 100 (32) | 108 (35) |
| IMDC risk group, n (%) |  |  |  |  |
| Favorable | 66 (20) | 62 (19) | 66 (21) | 58 (19) |
| Intermediate | 210 (64) | 214 (65) | 199 (63) | 198 (65) |
| Poor | 54 (16) | 52 (16) | 51 (16) | 49 (16) |
| MSKCC risk group, n (%) |  |  |  |  |
| Favorable | 150 (45) | 150 (46) | 144 (46) | 138 (45) |
| Intermediate | 139 (42) | 135 (41) | 131 (41) | 127 (42) |
| Poor | 41 (12) | 43 (13) | 41 (13) | 40 (13) |
| Metastatic site per IRC, n (%) |  |  |  |  |
| Lung | 204 (62) | 212 (65) | 196 (62) | 197 (65) |
| Liver | 88 (27) | 103 (31) | 85 (27) | 96 (31) |
| Bone | 77 (23) | 65 (20) | 72 (23) | 60 (20) |
| Lymph node | 206 (62) | 199 (61) | 196 (62) | 184 (60) |
| Brain | 2 (<1) | 1 (<1) | 2 (1) | 1 (<1) |
| Other | 23 (7) | 21 (6) | 21 (7) | 18 (6) |
| Prior VEGFR TKI therapy, n (%) |  |  |  |  |
| 1 | 235 (71) | 229 (70) | 226 (72) | 212 (70) |
| ≥2 | 95 (29) | 99 (30) | 90 (28) | 93 (30) |
| Prior nephrectomy, n (%) | 283 (86) | 279 (85) | 271 (86) | 258 (85) |

Demographics and baseline characteristics are shown in the METEOR study for all randomized patients and for those with baseline biomarker data; characteristics were similar in the two groups.

**Supplementary Table 2. Association between baseline biomarkers and IMDC risk groups**

|  | **Mean Baseline Biomarker Levels*** | | | **Values from ANOVA Analysis** | | | | |
| --- | --- | --- | --- | --- | --- | --- | --- | --- |
| **Plasma  Biomarker** | **Poor** | **Intermediate** | **Favorable** | **Poor (Intercept)** | **Intermediate** | **Favorable** | **F** | **P-value** |
| CA9 | 303.1 | 167.8 | 171.2 | 6.67 | -0.54 | -0.50 | 2.7 | 0.07 |
| HGF | 1724 | 1008 | 755.3 | 10.16 | -0.57 | -0.80 | 28.4 | <0.0001 |
| MET | 206.4 | 189.4 | 180.8 | 7.61 | -0.096 | -0.15 | 4.2 | 0.02 |
| GAS6 | 17210 | 15560 | 14890 | 14.01 | -0.15 | -0.20 | 5.8 | 0.003 |
| AXL | 16990 | 15400 | 14990 | 13.96 | -0.11 | -0.13 | 3.2 | 0.04 |
| VEGF | 38.84 | 20.04 | 18.12 | 4.23 | -1.10 | -1.50 | 16.7 | <0.0001 |
| VEGFR2 | 5028 | 4736 | 5029 | 12.26 | -0.10 | -0.01 | 4.4 | 0.01 |
| IL-8 | 12.41 | 8.371 | 5.847 | 2.52 | -0.29 | -0.60 | 4.3 | 0.01 |

ANOVA was performed on biomarker levels normalized by log_2_ transformation and all coefficients are on a log_2_ scale. The intercept represents the mean protein level in the poor risk group, and the intermediate and favorable columns show the difference in mean levels of those groups from the poor group. The F statistic represents the ratio of variance between risk groups and the variance within the risk groups; F statistic >1 indicates a difference between the means in the sample groups.

*All protein levels are shown in pg/mL except for MET, which is shown in ng/mL.

**Supplementary Table 3. Analysis of progression-free survival and overall survival for cabozantinib versus everolimus by baseline biomarker level dichotomized at the median**

| **Plasma Biomarker** | **Progression-Free Survival**  **HR (95% CI) for Cabozantinib vs Everolimus** | | **Overall Survival  HR (95% CI) for Cabozantinib vs Everolimus** | |
| --- | --- | --- | --- | --- |
|  | **≥median biomarker** | **<median biomarker** | **≥median biomarker** | **<median biomarker** |
| CA9 | 0.52 (0.39, 0.70) | 0.52 (0.39, 0.69) | 0.66 (0.48, 0.90) | 0.65 (0.47, 0.91) |
| HGF | 0.54 (0.41, 0.70) | 0.46 (0.34, 0.63) | 0.74 (0.56, 0.99) | 0.48 (0.32, 0.70) |
| MET | 0.48 (0.36, 0.65) | 0.55 (0.41, 0.74) | 0.62 (0.46, 0.84) | 0.67 (0.48, 0.94) |
| GAS6 | 0.57 (0.43, 0.75) | 0.47 (0.35, 0.63) | 0.76 (0.56, 1.02) | 0.53 (0.37, 0.75) |
| AXL | 0.65 (0.50, 0.86) | 0.39 (0.29, 0.53) | 0.78 (0.58, 1.06) | 0.54 (0.38, 0.76) |
| VEGF | 0.60 (0.46, 0.80) | 0.43 (0.32, 0.58) | 0.78 (0.58, 1.04) | 0.51 (0.36, 0.74) |
| VEGFR2 | 0.51 (0.38, 0.69) | 0.51 (0.38, 0.68) | 0.68 (0.49, 0.94) | 0.63 (0.46, 0.86) |
| IL-8 | 0.47 (0.35, 0.63) | 0.58 (0.43, 0.78) | 0.69 (0.51, 0.93) | 0.62 (0.43, 0.88) |

**Supplementary Table 4. Analyses of PFS and OS in each treatment arm based on continuous log_2_** of baseline biomarker level

| **Plasma Biomarker** | **Progression-Free Survival** | | | **Overall Survival** | | |
| --- | --- | --- | --- | --- | --- | --- |
|  | **Cabozantinib**  **HR (95% CI)** | **Everolimus**  **HR (95% CI)** | **P_interaction_** | **Cabozantinib**  **HR (95% CI)** | **Everolimus**  **HR (95% CI)** | **P_interaction_** |
| CA9 | 0.96 (0.90, 1.03) | 0.99 (0.93, 1.06) | 0.56 | 1.07  (0.98, 1.16) | 1.08  (1.01, 1.17)* | 0.86 |
| HGF | 1.36 (1.16, 1.61)* | 1.24 (1.09, 1.42)* | 0.57 | 1.54  (1.31, 1.80)* | 1.33  (1.16, 1.52)* | 0.16 |
| MET | 1.59 (1.07, 2.37)* | 1.48 (0.95, 2.29) | 0.89 | 2.59  (1.62, 4.14)* | 2.01  (1.23, 3.26)* | 0.48 |
| GAS6 | 1.42 (0.98, 2.07) | 1.23 (0.91, 1.66) | 0.64 | 2.63  (1.71, 4.05)* | 1.73  (1.23, 2.43)* | 0.12 |
| AXL | 1.77 (1.20, 2.62)* | 1.18 (0.81, 1.73) | 0.14 | 1.66  (1.04, 2.63)* | 1.59  (1.03, 2.45)* | 0.97 |
| VEGF | 1.08 (1.01, 1.16)* | 1.05 (0.98, 1.12) | 0.59 | 1.19  (1.09, 1.29)* | 1.14  (1.05, 1.23) | 0.49 |
| VEGFR2 | 0.74 (0.50, 1.10) | 1.11 (0.76, 1.62) | 0.16 | 0.85  (0.56, 1.28) | 0.90  (0.59, 1.36) | 0.83 |
| IL-8 | 1.10 (0.99, 1.23) | 1.10 (0.99, 1.21) | 0.92 | 1.26  (1.11, 1.42)* | 1.22  (1.10, 1.37) | 0.73 |

P-interaction was obtained from a separate model that included the interaction between treatment and biomarker level.

* p<0.05 for the analysis.

**Supplementary Table 5. Multivariable analyses of PFS and OS including IMDC risk group in each treatment arm based on continuous log_2_ of baseline biomarkers**

| **Plasma Biomarker** | **Progression-Free Survival**  **HR (95% CI)** | | | **Overall Survival  HR (95% CI)** | | |
| --- | --- | --- | --- | --- | --- | --- |
|  | **Cabozantinib** | **Everolimus** | **P_interaction_** | **Cabozantinib** | **Everolimus** | **P_interaction_** |
| HGF | 1.24 (1.03, 1.50)* | 1.21 (1.05, 1.39)* | 0.71 | 1.39  (1.16, 1.65)* | 1.24  (1.07, 1.43)* | 0.31 |
| MET | 1.44 (0.98, 2.13) | 1.38 (0.89, 2.16) | 0.81 | 2.25  (1.43, 3.55)* | 1.72  (1.06, 2.78)* | 0.42 |
| GAS6 | 1.40 (0.97, 2.04) | 1.19 (0.88, 1.62) | 0.55 | 2.47  (1.60, 3.80)* | 1.6  (1.13, 2.27)* | 0.098 |
| AXL | 1.60 (1.11, 2.33)* | 1.12 (0.76, 1.65) | 0.13 | 1.41  (0.92, 2.17) | 1.53  (0.99, 2.35) | 0.77 |
| VEGF | 1.06 (0.98, 1.13) | 1.04 (0.97, 1.11) | 0.58 | 1.14  (1.04, 1.24)* | 1.11  (1.03, 1.20)* | 0.65 |
| IL-8 | 1.08 (0.97, 1.20) | 1.09 (0.99, 1.20) | 0.86 | 1.22  (1.08, 1.37)* | 1.22  (1.10, 1.36)* | 0.98 |

Biomarkers were included in the multivariable analysis that had p<0.1 in at least one of the univariate analyses for PFS or OS by treatment arm. P-interaction was obtained from a separate model that included the interaction between treatment and biomarker level.

* p<0.05 for the analysis.

**Supplementary Table 6. Multivariable analyses of progression-free survival and overall survival in each treatment arm including baseline biomarker levels expressed as continuous variables and change in biomarkers at week 4 as covariates**

| **Plasma Biomarker** | **HR (95% CI)** | **P_value_** |
| --- | --- | --- |
| **Progression-Free Survival (Cabozantinib)** | | |
| AXL | 1.55 (1.01, 2.38) | 0.05 |
| HGF | 1.16 (0.90, 1.49) | 0.24 |
| VEGF | 1.05 (0.96-1.14) | 0.30 |
| ∆IL8 | 0.95 (0.85, 1.06) | 0.34 |
| ∆HGF | 0.89 (0.70, 1.13) | 0.35 |
| MET | 1.15 (0.75, 1.75) | 0.52 |
| IL8 | 0.97 (0.83, 1.13) | 0.69 |
| GAS6 | 1.04 (0.69, 1.58) | 0.84 |
| **Overall Survival (Cabozantinib)** | | |
| HGF | 1.45 (1.11, 1.90) | 0.006* |
| GAS6 | 2.04 (1.20, 3.46) | 0.009* |
| MET | 1.36 (0.82, 2.23) | 0.23 |
| VEGF | 1.07 (0.94, 1.22) | 0.30 |
| ∆CA9 | 0.95 (0.86-1.06) | 0.39 |
| ∆HGF | 1.08 (0.85, 1.38) | 0.52 |
| AXL | 1.14 (0.70, 1.83) | 0.60 |
| ∆GAS6 | 1.16 (0.66, 2.04) | 0.61 |
| IL8 | 1.05 (0.87, 1.26) | 0.62 |
| ∆VEGF | 0.98 (0.88, 1.09) | 0.72 |
| ∆IL8 | 1.01 (0.87, 1.16) | 0.92 |
| **Progression-Free Survival (Everolimus)** | | |
| HGF | 1.21 (1.05, 1.39) | 0.009* |
| MET | 1.36 (0.88, 2.09) | 0.17 |
| IL8 | 1.05 (0.95, 1.16) | 0.35 |
| **Overall Survival (Everolimus)** | | |
| HGF | 1.20 (1.01, 1.43) | 0.04* |
| GAS6 | 1.34 (0.88, 2.05) | 0.17 |
| IL8 | 1.09 (0.96, 1.24) | 0.19 |
| MET | 1.37 (0.81, 2.31) | 0.24 |
| VEGF | 1.05 (0.94, 1.16) | 0.40 |
| AXL | 1.12 (0.69, 1.83) | 0.65 |
| ∆VEGF | 0.99 (0.89, 1.11) | 0.91 |
| CA9 | 1.00 (0.91, 1.09) | 0.93 |
|  |  |  |

Biomarkers were included in the multivariable analysis, if p<0.10 in the univariate analyses. Hazard ratios are for high versus low biomarker levels. ∆ indicates the covariate is change in the biomarker at week 4; all other covariates are baseline biomarker levels expressed as continuous variables.

* p<0.05 for the analysis.
